# Exploring the nutritional riches of pointed gourd (*Trichosanthes dioica* Roxb.): a multivariate approach for selection of nutritionally superior genotypes for breeding programmes

**DOI:** 10.3389/fpls.2026.1865400

**Published:** 2026-07-15

**Authors:** Ankit Kumar Sinha, Bhavana P., Arun Kumar Singh, Jeetendra Kumar Ranjan, Harshwardhan Choudhary, Gyan Prakash Mishra, Reshma Shinde, Mahesh Kumar Dhakar, Paresh Chaukhande, Neha Singh, Devireddy Meghana, V Madhumathi, Sajiya Ekbal, Anup Das

**Affiliations:** 1 ICAR-Indian Agricultural Research Institute, New Delhi, India; 2 ICAR-Research Complex for Eastern Region, FSRCHPR, Ranchi, Jharkhand, India; 3 Birsa Agricultural University-Horticultural College, Khutpani, Ranchi, India; 4 ICAR- Indian Institute of Horticultural Research, Bengaluru, India; 5 ICAR- Research Complex for Eastern Region, Patna, India

**Keywords:** antioxidant activity, nutritional quality, pointed gourd, principal component analysis, shelf-life

## Abstract

Pointed gourd (*Trichosanthes dioica* Roxb.) is an underutilized yet nutritionally rich vegetable with significant potential to address micronutrient deficiencies in India. However, limited information is available on the extent of variability in nutritional and antioxidant traits and their interrelationships among diverse pointed gourd genotypes, restricting the identification of nutritionally superior breeding materials. A total of 46 genotypes were evaluated for 15 fruit quality parameters, including total phenols, ascorbic acid, carbohydrates, protein, essential minerals, moisture content, antioxidant capacity, shelf-life and pericarp thickness, to identify nutritionally superior genotypes with improved shelf-life and transportability, suitable for long-distance markets and efficient supply chain management. Among the genotypes, HAP-79 exhibited the longest shelf-life (6 days) along with high antioxidant potential (154.39 mg/100g) and nutrient content e.g., zinc (Zn), iron (Fe), copper (Cu). Substantial variability was observed among genotypes, indicating strong genetic diversity for nutritional traits. Antioxidant activity showed positive association with phenolic content and vitamin C, while protein content exhibited strong linkage with mineral accumulation, suggesting the possibility of simultaneous improvement of multiple nutritional parameters. Principal component analysis (PCA) revealed an inverse relationship between moisture and protein content, while calcium and iron showed close alignment. Genotypes such as HAP-35, HAP-6, HAP-79, HAP-115, HAP-18, HAP-117, HAP-106, HAPH-1, Swarna Alaukik and Swarna Rekha exhibited superior nutrient accumulation, highlighting their potential for biofortification. Hierarchical clustering grouped genotypes into four distinct clusters, with Swarna Suruchi and Swarna Rekha clustering together, while Swarna Alaukik formed a separate group. These findings provide valuable insights for selecting nutritionally enriched genotypes for future breeding programs.

## Graphical Abstract

**Figure:**
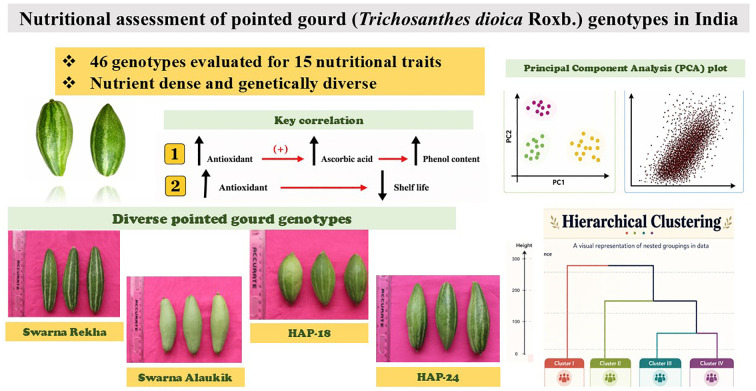

## 1 Introduction

In India, addressing malnutrition and micronutrient deficiencies continues to be an important public health priority, especially for supporting the well-being of vulnerable populations (Kotecha, 2008; Haridas et al., 2022). Incorporating nutrient-dense vegetables like pointed gourd (*Trichosanthes dioica* Roxb.), commonly known as *parwal* or *potol*, into daily diets can play a crucial role in addressing these nutritional gaps. *Trichosanthes dioica* Roxb., is a perennial, dioecious climbing herb that belongs to the Cucurbitaceae family. The chromosome number of pointed gourd is 2n=2x=22, with pollen mother cells containing 11 bivalents (Sinha et al., 2007; Hassan et al., 2022b). This plant is native to the Assam-Bengal regions of India. Pointed gourd is cultivated extensively in India, Bangladesh and Nepal, with a diverse range of genotypes exhibiting variation in shape, size, biochemical composition and yield potential. India is the largest producer of pointed gourd, occupying nearly 77,000 ha with an annual production of approximately 1.038 million tonnes (Ministry of Agriculture & Farmers Welfare, 2025). It is extensively grown in several states of India and occupies a prominent place in the food history of eastern India, particularly in West Bengal, Bihar, Uttar Pradesh and Orissa (Bharathi and Vishalnath, 2011; Hassan and Miyajima, 2019; Sinha et al., 2025). Its immature fruits have long been consumed in diverse traditional preparations, ranging from curries and stir-fries to stuffed dishes such as *dorma* (stuffed gourd with roe or minced filling) (Vyas and Sharma, 2022). In addition to the fruits, tender shoots and leaves are also utilized as food and in traditional household remedies (Gupta et al., 2012). The crop is recognized in ancient Ayurvedic literature, including the *Charaka Samhita*, for its therapeutic dietary value (Gupta et al., 2012; Vyas and Sharma, 2022). Compared to other cucurbits, pointed gourd stands out for its higher nutritional content (Pandit and Hazra, 2008) and due to which, it is popularly called as ‘king of gourds’ (Yadav et al., 2022). Fruits of pointed gourd are especially high in nutrients; they have ten times more protein than bottle gourd and four times more vitamin A (153 mg/100g) than ridge, snake and ash gourds (Gopalan et al., 1989). Furthermore, as noted by Choudhury in 1996, fresh pointed gourd fruits are an excellent source of vital minerals, such as phosphorous, calcium, magnesium, sodium, potassium, copper, sulphur and chlorine which play significant roles in various physiological processes. Additionally, pointed gourd contains high levels of total soluble solids (TSS), carbohydrates, proteins, phenols, flavonoids and ascorbic acid, which contribute to its functional and nutritional properties.

Pointed gourd (*Trichosanthes dioica* Roxb.), an important cucurbitaceous vegetable widely cultivated in India, occupies a significant place in traditional Ayurvedic medicine owing to its diverse therapeutic properties (Sinha et al., 2025). Various parts of the plant, particularly the fruits and leaves, have traditionally been used as febrifuges, liver tonics and remedies for jaundice, alcoholism, intestinal parasitic infections and inflammatory conditions associated with respiratory and rheumatic disorders (Alam et al., 2011; Gupta et al., 2012; Vyas and Sharma, 2022). The fruits exhibit antipyretic, anthelmintic, expectorant, anti-rheumatic, antihypertensive, and cardioprotective activities, contributing to improved circulatory health (Alam et al., 2011; Khandaker et al., 2018; Vyas and Sharma, 2022). These pharmacological effects are largely attributed to the presence of bioactive compounds such as flavonoids, triterpenes (particularly cucurbitacin B), trichosanthin, tannins, saponins and alkaloids, which possess antioxidant, anti-inflammatory, lipid-modulating and other health-promoting properties (Gupta et al., 2012; Patel and Shah, 2013; Khandaker et al., 2018). Furthermore, these phytochemicals contribute to the therapeutic potential of pointed gourd without exhibiting acute toxicity even at relatively higher doses (Shah and Seth, 2010; Khandaker et al., 2018).

Pointed gourd has been widely studied for its medicinal properties. Research indicates that its bioactive compounds exhibit anti-diabetic, antimicrobial and hepatoprotective effects. Studies have also highlighted its role in glycemic control, where aqueous extracts of *T. dioica* leaves significantly reduced blood glucose levels in diabetic models (Sharma et al., 2023). The presence of bioactive peptides further enhances its therapeutic potential, particularly in managing metabolic disorders (Khandaker et al., 2018). Moreover, experimental studies have shown that *T. dioica* exhibits neuroprotective properties, indicating its potential role in preventing age-related cognitive decline (Hassan et al., 2022a).

Despite its nutritional significance, limited research has been conducted to explore the variability in biochemical compounds and antioxidant potential among different pointed gourd genotypes. Previous studies have primarily focused on morphological characteristics, yield performance (Sinha et al., 2024; Dhaker et al., 2025), and a limited number of quality traits, leaving comprehensive information on biochemical composition, antioxidant activity, mineral content, and their interrelationships largely unexplored. Furthermore, the extent of genetic variability for these nutritionally important traits among pointed gourd germplasm remains poorly understood. Such knowledge is critical for identifying superior genotypes and developing breeding strategies aimed at enhancing nutritional quality. Therefore, the present study evaluated a diverse set of pointed gourd genotypes, including released varieties, for key biochemical, antioxidant, and mineral traits. In addition, principal component analysis (PCA) and agglomerative hierarchical clustering (AHC) were employed to assess trait associations, characterize genetic diversity, and identify nutritionally superior genotypes. The findings provide valuable information for nutritional improvement and the development of nutrient-rich pointed gourd cultivars adapted to diverse agroecological conditions.

## 2 Materials and methods

**Figure 1:**
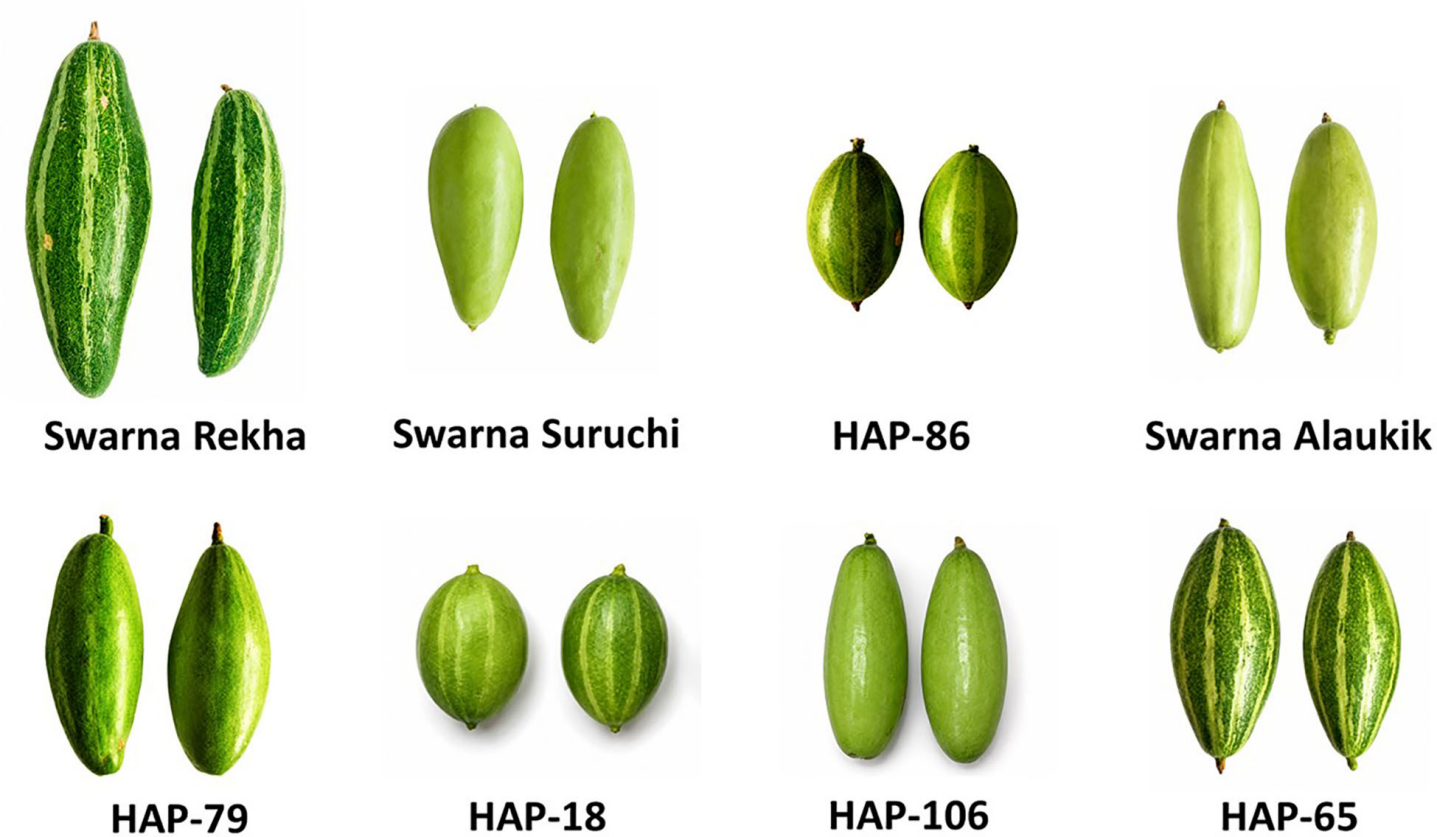
Representative fruits of released varieties and selected pointed gourd genotypes showing variation in fruit morphology.

The experiment was conducted at Farm-I of the ICAR–Research Complex for Eastern Region (RCER), Farming Systems Research Centre for Hill and Plateau Region (FSRCHPR), Ranchi, Jharkhand, India. The research site is located at an average altitude of approximately 625 meters (2,064 feet) above mean sea level and at coordinate of 23° 17’ 6.3″ N latitude and 85° 24’ 41.3″ E longitude. The research farm is situated within the tropical to sub-tropical climatic zone. The research field has been identified as having laterite soil with a sandy loam texture on the surface and the soil pH ranges from 4.5 to 5.5. In February 2021, vine cuttings were transplanted into the field following a Randomized Block Design (RBD) with three replications. Each experimental plot measured 4 m × 2 m and accommodated 12 plants, which were spaced at 2 m between rows and 1 m between plants within a row. As pointed gourd is a perennial crop, the established plants were maintained under recommended agronomic practices throughout the study period. During the first year, special care was taken to ensure proper establishment of the vegetatively propagated plants. Following the winter dormancy period, the plants resumed active growth, flowered, and set fruits normally. Observations on nutritional and biochemical traits were subsequently recorded during the 2022 growing season. The experimental material included 46 pointed gourd genotypes (**Supplementary Table 1**), among which were three varieties - Swarna Rekha (SR), Swarna Suruchi (SS) and Swarna Alaukik (SA) developed by the Centre, two hybrids (HAPH-1 and HAPH-2), and 41 genotypes (HAP series) selected from germplasm collected from the eastern plateau and hill region of India. The genotypes exhibited substantial diversity in fruit characteristics, including colour intensity, striping pattern, fruit length, diameter, and shape. Fruits ranged from light green to dark green with varying degrees of longitudinal striping. Representative fruit types of some genotypes are shown in **Figure 1**. For each genotype, observations were recorded from five randomly selected fruits per replication (at green, tender immature stage) across three replications. The sampled fruits were collected from multiple plants within each replication to ensure representative sampling of the genotype.

### 2.1 Estimation of biochemical constituents

Biochemical and nutritional parameters were evaluated following well-established analytical protocols. Appropriate reference standards, calibration solutions, and standard curves were employed for quantitative estimations wherever applicable, as specified in the respective methods.

#### 2.1.1 Shelf-life of fruits

The shelf-life of fruits was assessed using the Physiological Loss in Weight (PLW) method under ambient room temperature conditions (25–30 °C). Initially, the fruits were weighed and their weight was recorded daily until it decreased by 10% from the initial weight. This helped determine the duration for which the fruits could be stored without significant weight loss.

#### 2.1.2 Pericarp thickness

Pericarp thickness (cm) was determined at the equatorial (middle) region of five randomly selected fruits per replication at the marketable stage using a digital vernier caliper (Model 500-196-30, Mitutoyo Corporation, Kanagawa, Japan), and the mean value was calculated and expressed in centimeters.

#### 2.1.3 Assessment of fruit shape and pubescence

Fruit shape and fruit pubescence were recorded at the marketable stage through visual observation of fruits from each genotype. Fruit shape was classified into seven categories, namely club-shaped, cylindrical, elongated spindle, oval, spheroidal, spindle, and spindle tapering, according to distinct morphological characteristics of fruit length, width, and tapering pattern. Fruit pubescence was recorded as either present or absent based on the occurrence of visible hairs on the fruit surface.

#### 2.1.4 Total soluble solid

Total Soluble Solids (TSS) is an important parameter for evaluating fruit quality, primarily indicating sweetness. In fruit juices, TSS mainly consists of sugars, along with organic acids, amino acids and soluble pectin. Total soluble solids (TSS) were measured using a hand-held refractometer (R3000-2, ATAGO Co., Ltd., Tokyo, Japan) and expressed as °Brix, which determines the refractive index of light passing through fruit juice. It was assessed at the marketable stage using five randomly selected fruits per replication. The juice was extracted by cutting, scraping and squeezing the pulp. One to two drops of clear juice were placed on a hand-held refractometer to determine TSS in °Brix. The average TSS value of individual fruits was recorded for each replication.

#### 2.1.5 Moisture percentage

The moisture content of the sample was determined by the gravimetric method by recording the sample weight before and after oven drying at 105 ± 2 °C until constant weight. The moisture content was calculated using the following formula:


$$\text{Moisture content}(\%)=\frac{[\text{Initial fresh weight }(\text{g})-\text{final dry weight }(\text{g})]\times 100}{\text{Initial fresh weight }(\text{g})}$$

#### 2.1.6 Total carbohydrate

The modified Anthrone method (Hodge and Hofreiter, 1962) was used to estimate total carbohydrate content. Carbohydrates were hydrolyzed with dilute HCl into simpler sugars and glucose was dehydrated in an acidic environment to form hydroxyl methyl furfural. This compound reacted with anthrone, producing a green-coloured complex with maximum absorbance at 630 nm. The chemicals used included 25% HCl, 95% ice-cold sulfuric acid, anthrone, sodium carbonate and D-glucose. A 100 mg ground fruit sample was hydrolyzed with 5 ml of 25% HCl in a boiling water bath for 3.5 hours. The solution was then neutralized with sodium carbonate, diluted to 100 ml with distilled water and centrifuged. A 0.5 ml aliquot was taken, mixed with 4 ml of Anthrone reagent and 0.5 ml distilled water, and heated in a boiling water bath for 8 minutes. After rapid cooling, absorbance was measured at 630 nm using a UV spectrophotometer and the total carbohydrate content was calculated using standard graph.

#### 2.1.7 Total protein

Total nitrogen in the fruit samples was estimated using a modified Kjeldahl method (Jackson, 1973). A 0.5 g portion of the powdered, defatted sample was mixed with 5 ml of the digestion mixture and a blank. Then, 10 ml of concentrated sulfuric acid (H_2_SO_4_) was added and the sample was digested in a Kjeldahl unit until the color changed from sky blue to green. After digestion, 20 ml of distilled water was added and mixed thoroughly. The mixture was then subjected to distillation and the extract was titrated against 0.1 N H_2_SO_4_ to determine nitrogen content. The total protein content was calculated by multiplying the nitrogen content by a conversion factor of 6.25. Finally, the protein content was adjusted from dry weight to fresh weight using the moisture percentage of each sample and expressed in g/100g of fruit.

#### 2.1.8 Ascorbic acid

The ascorbic acid content in pointed gourd fruit was estimated using the 2,6-Dichlorophenol indophenol visual titration method, as described by Ranganna (1986). This method relies on the reduction of 2,6-Dichlorophenol indophenol dye to a colorless leuco-base, while ascorbic acid in the sample is oxidized to dehydroascorbic acid, producing a pink endpoint. Oxalic acid served as the titration medium. For dye preparation, 21 mg of sodium bicarbonate and 25 mg of 2,6-Dichlorophenol indophenol were dissolved in 80 ml of heated, cooled distilled water, with the final volume adjusted to 100 ml. For sample extraction, a 5 g portion of fresh green fruits was accurately weighed and macerated with 10 ml of a 4% oxalic acid solution. The mixture was then filtered and the filtrate was diluted to a total volume of 100 ml. During titration, 5 ml of the prepared extract was transferred into a conical flask containing 10 ml of a 4% oxalic acid solution. The dye solution was gradually added until a stable pink color persisted for a few minutes, indicating the endpoint. The volume of dye required for this color change was recorded as the titre value. The ascorbic acid content in the sample was determined using the slope of the standard curve and expressed as milligrams per 100 g of fresh weight.

#### 2.1.9 Total phenols

The total phenolic content was estimated using a spectrophotometric method, following the protocol established by Singleton et al. (1999). The analysis utilized Folin–Ciocalteu reagent (FCR) to assess the phenolic compounds present in the sample. 5 g fruit sample was ground using a mortar and pestle with 20 ml of 80% methanol (CH_3_OH), repeating the extraction process two to three times. The homogenized mixture was then filtered Using Whatman filter paper No. 1 and the final volume of the extract was adjusted to 50 ml. For analysis, 0.5 ml of the extract was transferred to a test tube, followed by the addition of 0.2 ml of 1N Folin–Ciocalteu reagent (FCR) and 3.3 ml of distilled water, ensuring thorough mixing. After allowing the mixture to incubate for 2 minutes, 1 ml of 20% sodium carbonate solution was added. The reaction was then left to incubate at room temperature for 30 minutes in darkness. The developed pink colour complex was measured at 765 nm using a spectrophotometer. A standard calibration curve was prepared using gallic acid and the results were expressed in terms of gallic acid equivalent (mg GAE/100 g fresh weight).

#### 2.1.10 Total antioxidant activity

The FRAP assay was performed following the method of Benzie and Strain (1996), based on the reduction of the ferric tripyridyl triazine [Fe (TPTZ)] ^3+^ complex to its ferrous [Fe (TPTZ)] ^2+^ form, producing a blue color with maximum absorbance at 593 nm. A 5 g plant sample was ground with 80% methanol and extracted multiple times into a 50 ml volumetric flask, with the final volume adjusted using 80% ethanol. For analysis, 0.2 ml of the extract was mixed with 1.8 ml of FRAP reagent, consisting of 0.1 M acetate buffer (pH 3.6), 10 mM TPTZ in 40 mM HCl, and 20 mM FeCl_3_·6H_2_O in a 10:1:1 ratio. The mixture was incubated at room temperature for 30 minutes and absorbance was measured at 593 nm using a spectrophotometer. Ascorbic acid standards were used as a reference and results were expressed as mg AEAC per 100 g of fresh weight.

#### 2.1.11 Estimation of essential nutrients (Zn, Fe, Cu, Mn, Ca and Mg)

The micronutrient content in the fruit was analyzed using Atomic Absorption Spectrophotometry, following the method of Lindsay and Norvell (1978). A 1 g fruit sample was digested with a 10 ml diacid mixture (nitric acid and perchloric acid in a 9:4 ratio) in a 100 ml beaker by heating at 200 °C until the solution turned from brown to ash-colored. The digested solution was washed with distilled water, filtered through Whatman No. 1 filter paper and transferred to a 100 ml volumetric flask, with the final volume adjusted using distilled water. The diacid extract was analyzed for zinc (Zn), copper (Cu), manganese (Mn), iron (Fe), calcium (Ca) and magnesium (Mg) using an Atomic Absorption Spectrophotometer. The micronutrient content, initially measured in ppm on a dry weight basis, was converted to mg/100 g fresh weight using the sample’s moisture content.

## 3 Statistical analysis

**Figure 2:**
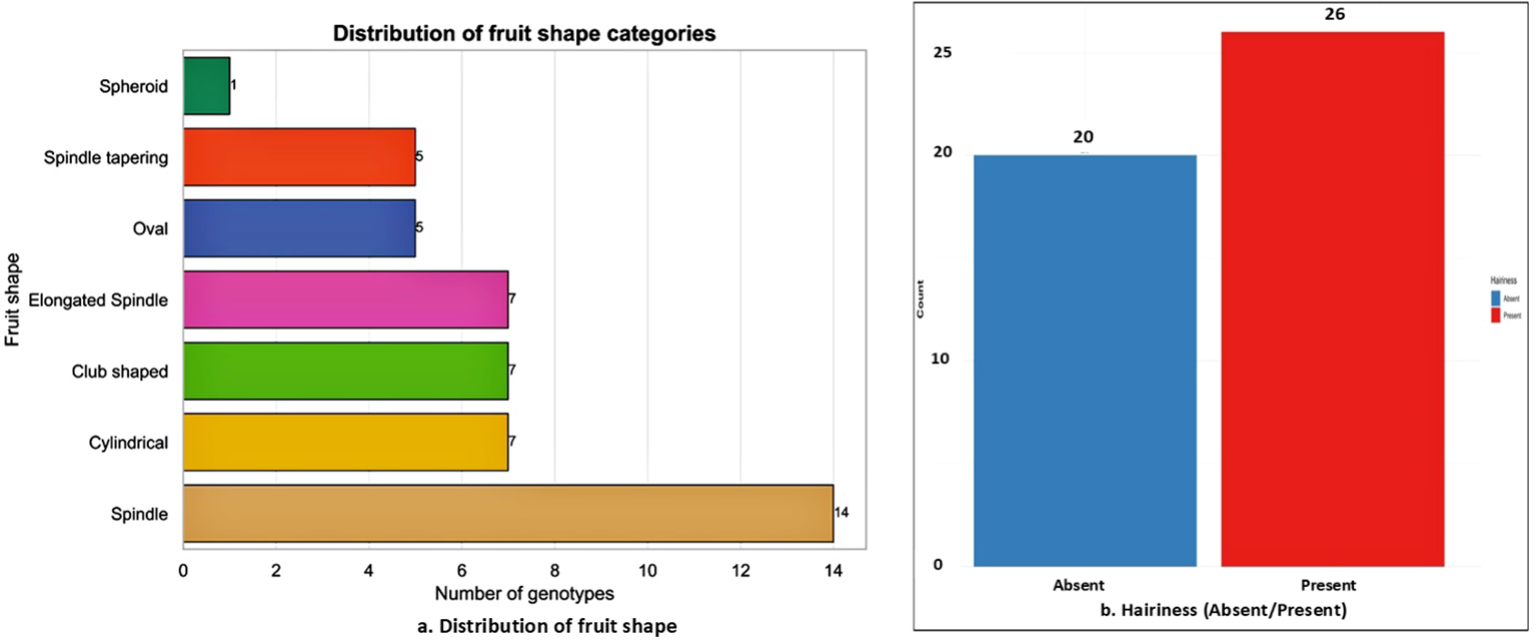
Distribution of fruit shape **(a)** and fruit pubescence/hairiness **(b)** among the studied genotypes.

**Figure 3:**
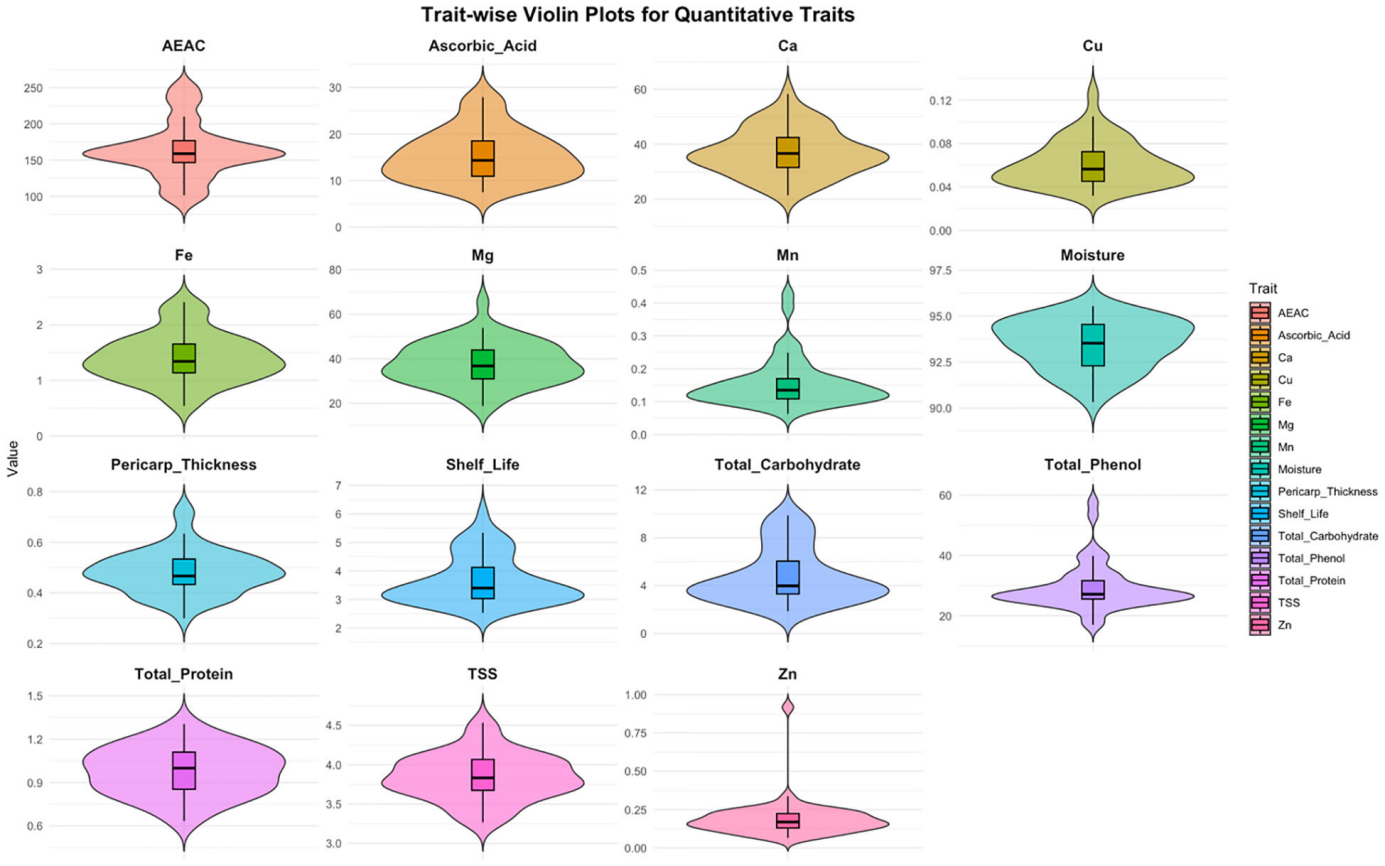
Distribution of quantitative traits among the diverse pointed gourd genotypes.

**Figure 4:**
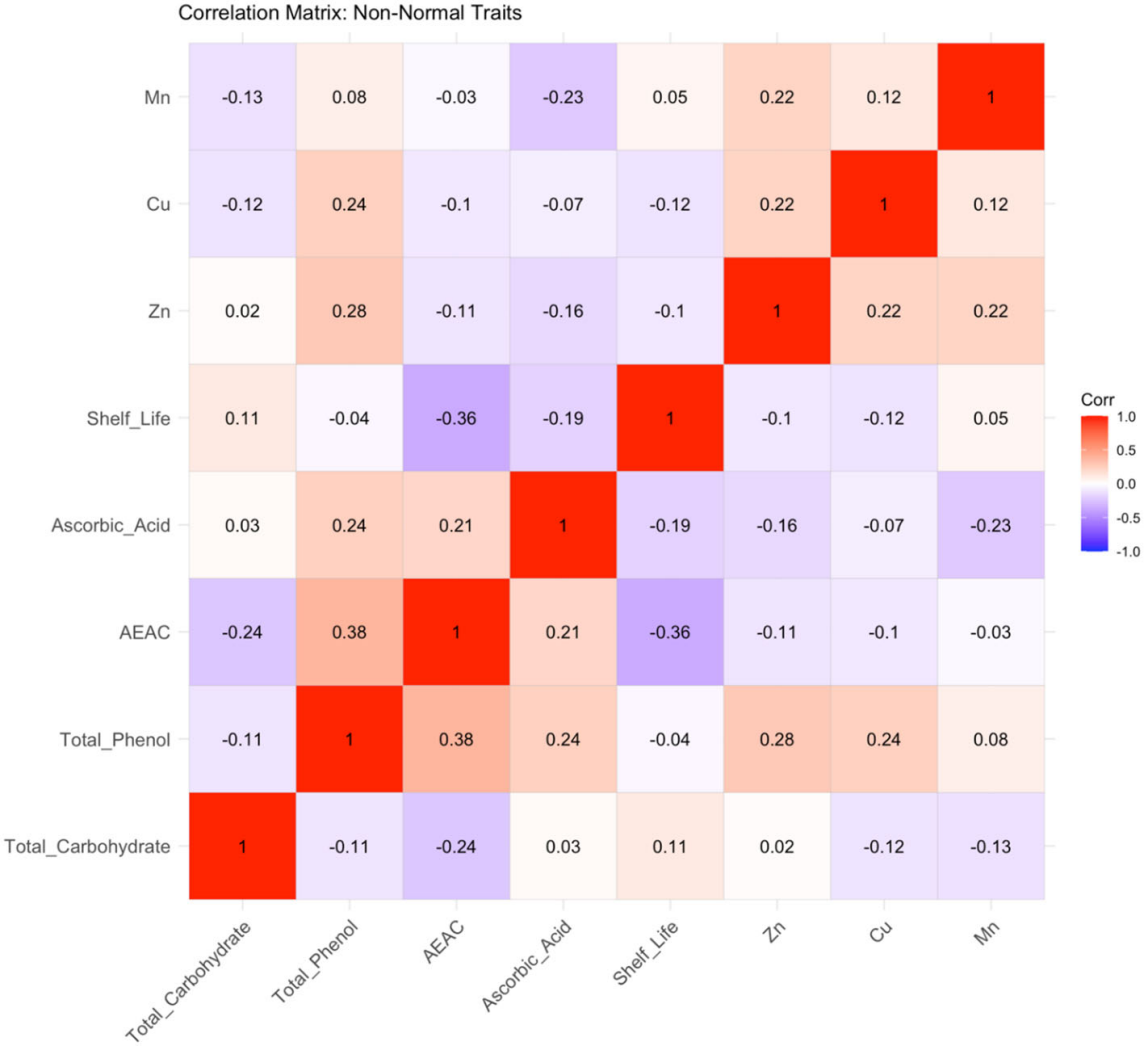
Correlation matrix of non-normal traits.

**Figure 5:**
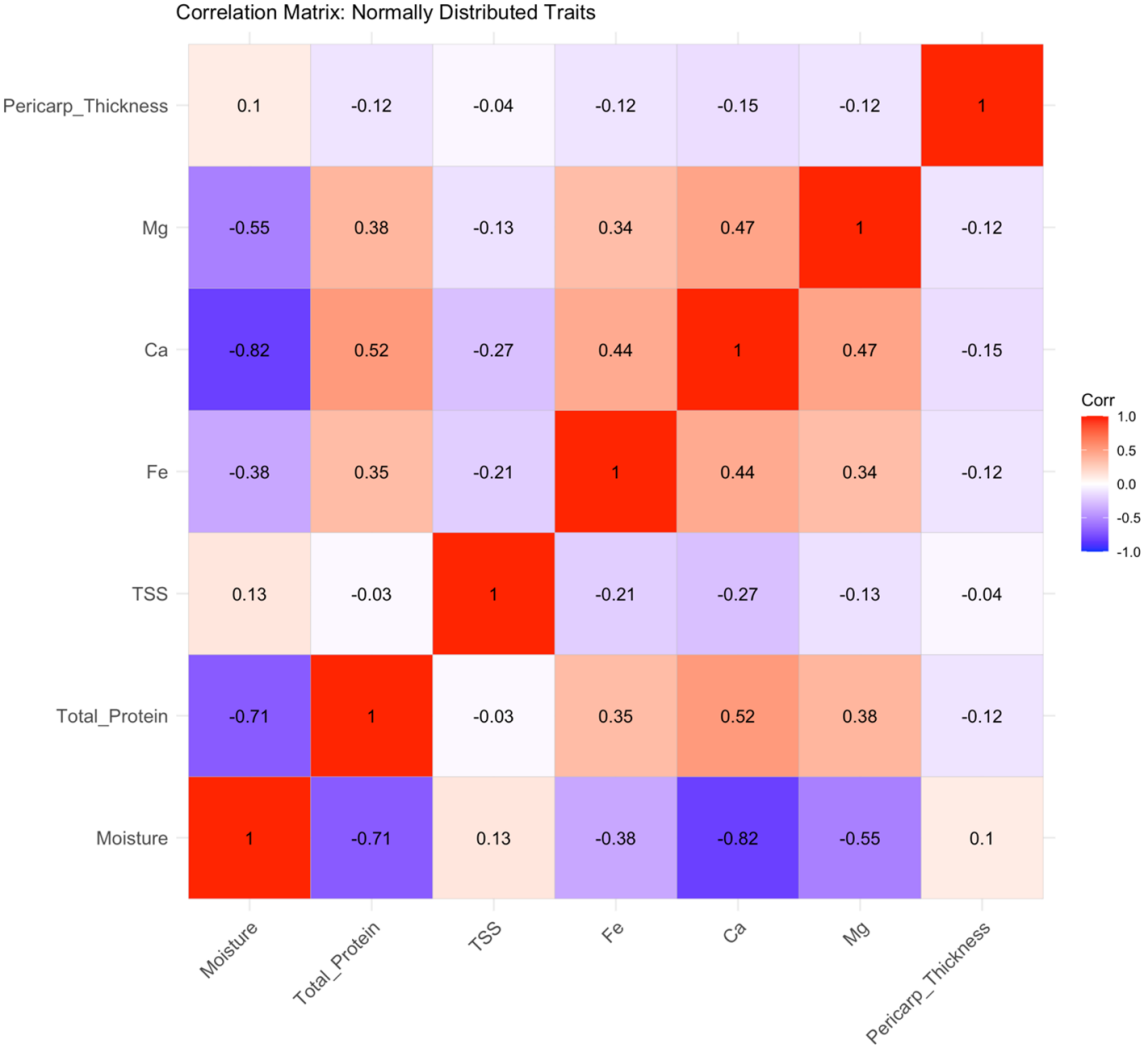
Correlation matrix of normally distributed traits.

The genetic assessment of pointed gourd genotypes was executed using RStudio Version 2023.09.1+494 (R Core Team, 2024), integrating statistical and graphical methodologies to examine both quantitative and qualitative attributes. Statistical significance was evaluated at P ≤ 0.05 and P ≤ 0.01, representing significant and highly significant differences, respectively. Normality of key traits, including moisture, total protein, TSS, Zn, Ca, Mg, and pericarp thickness, was evaluated using the Shapiro–Wilk test to determine the appropriate correlation model (**Table 1**). Descriptive statistics, including mean, standard deviation, coefficient of variation, and range, were computed (**Table 1**). Categorical traits, such as fruit shape and pubescence, were analyzed using bar plots (**Figures 2a, b**), while violin plots generated using ggplot2 captured data distribution, density, and outliers (**Figure 3**) (Wickham et al., 2023). Pearson’s correlation was applied to normally distributed traits, whereas Spearman’s correlation was utilized for non-parametric data. Correlation matrices were visualized through heatmaps using ggcorrplot and corrplot, facilitating the identification of trait interrelationships (Alboukadel, 2017; Wei & Simko, 2021) (**Figures 4**, **5**). For correlation analysis, the sample size was N = 46, and correlation significance was evaluated at P ≤ 0.05 and P ≤ 0.01, represented by * and **, respectively. Weak and non-significant correlations were not considered for biological interpretation.

**Table 1:** Mean performance and variability analysis of diverse pointed gourd genotypes for 15 qualitative characters.

Sl. No.	Genotypes	Shelf-life of fruit (days)	Pericarp thickness (cm)	TSS (°Brix)	Moisture (%)	Total carbohydrate (%)	Total protein (g/100 g)	Ascorbic acid (mg/100 g)	Total phenol (mg GAE/100 g)	Antioxidant activity (mg AEAC/100 g)	Zn (mg/100 g fresh wt.)	Fe (mg/100 g fresh wt.)	Cu (mg/100 g fresh wt.)	Mn (mg/100 g fresh wt.)	Ca (mg/100 g fresh wt.)	Mg (mg/100 g fresh wt.)
1	Swarna Rekha (SR)	3.87	0.50	3.73	94.29	2.93	1.12	12.91	57.76	251.57	0.22	1.40	0.05	0.10	35.04	36.18
2	Swarna Suruchi (SS)	3.00	0.50	3.47	93.47	3.64	0.77	11.39	53.15	204.34	0.24	1.33	0.13	0.17	41.87	43.27
3	Swarna Alaukik (SA)	2.73	0.47	3.33	93.05	3.43	0.98	9.02	34.47	152.72	0.34	1.47	0.09	0.38	46.80	49.56
4	HAP-2	2.83	0.53	3.57	94.83	1.86	0.73	9.47	28.93	177.11	0.20	2.13	0.06	0.17	33.95	34.45
5	HAP-6	3.10	0.40	3.37	92.27	4.44	0.89	10.61	27.33	161.94	0.22	2.17	0.09	0.20	50.59	53.82
6	HAP-8	3.40	0.30	3.70	92.26	9.53	1.07	19.64	32.87	100.47	0.29	1.64	0.08	0.12	36.68	51.81
7	HAP-18	3.50	0.37	3.67	92.26	5.03	1.30	15.17	29.49	108.95	0.25	1.81	0.07	0.13	47.49	37.25
8	HAP-23	3.43	0.47	3.47	94.91	3.20	0.81	14.29	26.67	129.36	0.17	1.35	0.05	0.12	22.91	28.10
9	HAP-24	4.77	0.57	3.93	95.54	9.15	0.80	12.36	25.36	101.68	0.15	1.18	0.04	0.13	26.47	43.94
10	HAP-28	3.40	0.47	3.77	94.47	8.24	0.96	14.38	26.86	129.99	0.08	1.25	0.07	0.12	37.62	37.07
11	HAP-35	5.33	0.50	3.83	94.17	4.38	1.00	8.50	26.96	105.37	0.17	1.78	0.04	0.22	34.68	65.46
12	HAP-38	3.23	0.53	3.83	93.11	3.71	1.11	9.82	23.86	126.94	0.24	1.18	0.07	0.13	38.16	45.89
13	HAP-40	3.83	0.43	4.00	93.30	6.97	1.07	18.76	25.92	151.68	0.15	1.03	0.05	0.23	40.84	43.45
14	HAP-41	4.47	0.40	3.87	95.29	2.83	0.63	16.22	23.39	91.24	0.07	1.25	0.07	0.14	30.88	27.93
15	HAP-45	3.00	0.47	4.17	94.34	5.47	0.85	13.94	25.46	162.12	0.22	1.79	0.05	0.15	26.09	47.04
16	HAP-63	4.63	0.37	4.53	93.01	8.25	0.92	7.51	23.30	141.70	0.23	1.25	0.11	0.17	35.94	43.80
17	HAP-65	4.10	0.53	4.07	95.38	3.68	0.64	12.79	26.21	151.74	0.16	1.03	0.08	0.10	28.38	18.77
18	HAP-70	3.00	0.47	4.40	93.22	3.70	1.07	19.59	25.74	127.46	0.20	2.31	0.04	0.14	38.68	32.22
19	HAP-72	4.47	0.70	3.60	93.58	2.41	0.84	10.76	25.74	152.72	0.16	1.56	0.04	0.28	44.42	37.13
20	HAP-74	3.10	0.47	4.03	94.90	2.23	0.91	17.56	26.21	166.38	0.11	1.32	0.09	0.14	30.09	28.40
21	HAP-75	3.20	0.40	4.13	95.04	2.13	0.84	19.39	25.36	152.72	0.20	1.23	0.09	0.08	36.70	30.49
22	HAP-76	3.47	0.60	3.70	92.35	4.14	1.20	27.61	26.11	164.14	0.17	1.66	0.05	0.09	35.09	49.00
23	HAP-77	3.57	0.53	4.07	94.21	4.37	1.02	12.49	23.67	160.73	0.07	1.12	0.04	0.09	36.86	23.12
24	HAP-78	4.13	0.37	4.00	93.90	9.86	1.03	9.74	23.95	120.31	0.12	0.94	0.05	0.12	27.37	33.01
25	HAP-79	6.00	0.53	4.13	93.37	4.09	1.15	9.85	26.11	154.39	0.22	1.43	0.06	0.17	32.21	36.44
26	HAP-81	3.73	0.47	4.10	93.18	7.06	1.00	9.44	27.43	165.69	0.17	0.99	0.06	0.12	36.63	35.19
27	HAP-85	5.13	0.37	4.07	94.77	7.75	0.90	15.23	28.27	154.50	0.12	0.98	0.05	0.11	30.09	27.95
28	HAP-86	5.13	0.53	4.47	93.35	5.21	1.16	16.55	28.46	148.68	0.12	0.92	0.07	0.13	34.23	41.65
29	HAP-88	3.03	0.57	4.23	91.69	2.35	1.11	21.42	27.61	167.88	0.19	1.59	0.08	0.11	46.46	45.06
30	HAP-92	2.83	0.73	3.83	94.05	2.37	0.86	8.93	26.11	176.53	0.09	0.65	0.06	0.15	26.39	30.64
31	HAP-94	3.03	0.63	4.07	94.60	3.62	0.74	12.28	17.57	183.58	0.08	0.95	0.04	0.09	21.44	30.51
32	HAP-95	2.83	0.37	3.67	93.94	6.24	1.04	17.08	20.10	159.53	0.25	1.57	0.04	0.15	32.85	34.79
33	HAP-96	3.20	0.57	3.30	94.58	4.82	0.89	26.10	17.01	168.11	0.14	0.68	0.04	0.15	31.36	32.10
34	HAP-102	3.53	0.50	3.70	90.97	5.35	1.00	13.05	30.15	237.97	0.27	1.23	0.06	0.10	50.36	50.21
35	HAP-106	3.00	0.50	3.73	91.35	6.51	1.28	27.91	39.45	250.12	0.09	1.58	0.06	0.16	41.46	42.65
36	HAP-110	2.73	0.37	3.93	91.37	9.78	0.91	25.96	34.47	229.91	0.18	0.97	0.06	0.17	50.50	48.47
37	HAP-111	3.03	0.47	3.53	93.08	3.80	1.03	10.55	23.86	231.53	0.14	1.54	0.05	0.21	42.67	33.61
38	HAP-112	4.70	0.43	4.07	95.04	2.58	0.74	17.08	39.82	203.65	0.18	0.54	0.03	0.18	24.89	27.16
39	HAP-113	5.03	0.43	3.80	92.14	3.97	1.11	17.35	38.60	210.05	0.13	1.79	0.05	0.12	37.05	39.85
40	HAP-114	2.53	0.53	4.00	94.90	8.57	0.80	17.77	27.90	157.98	0.16	1.22	0.04	0.06	33.17	19.33
41	HAP-115	3.00	0.47	3.70	90.33	3.76	1.19	21.66	30.06	178.05	0.10	1.29	0.09	0.10	58.27	40.34
42	HAP-116	3.13	0.47	3.83	94.06	3.33	0.89	21.80	34.38	182.20	0.14	1.85	0.04	0.11	36.93	31.92
43	HAP-117	3.40	0.57	3.73	91.14	2.82	1.18	16.24	28.37	158.50	0.14	2.41	0.07	0.27	47.31	35.83
44	HAPEL-1	3.13	0.43	3.80	92.00	3.99	1.19	12.63	32.12	146.04	0.25	2.31	0.05	0.11	51.20	47.06
45	HAPH-1	5.07	0.50	3.67	91.92	3.37	1.20	13.61	32.59	158.15	0.24	1.70	0.07	0.43	43.23	39.31
46	HAPH-2	3.70	0.43	3.27	94.38	3.29	1.04	22.08	41.98	164.72	0.25	1.43	0.05	0.25	33.26	25.88
	*Mean*	3.66	0.48	3.84	93.46	4.79	0.98	15.40	29.29	162.63	0.19	1.41	0.06	0.16	37.08	37.76
	*CV%*	9.61	18.09	7.11	1.24	6.52	7.79	7.51	5.33	3.86	3.22	2.54	2.88	2.40	2.52	2.88
	*SeM ±*	0.20	0.01	0.16	0.67	0.18	0.04	0.67	0.90	3.62	0.00	0.02	0.00	0.00	0.54	0.63
	*CD at 5%*	0.57	0.11	0.44	1.88	0.51	0.12	1.88	2.53	10.18	0.01	0.06	0.00	0.01	1.51	1.77
	*CD at 1%*	0.76	0.15	0.59	2.48	0.67	0.16	2.48	3.35	13.48	0.01	0.08	0.00	0.01	2.01	2.34
	*Minimum*	2.53	0.30	3.27	90.33	1.86	0.63	7.51	17.00	91.24	0.07	0.54	0.03	0.06	21.44	18.77
	*Maximum*	6.00	0.73	4.53	95.54	9.86	1.30	27.91	57.76	251.57	0.34	2.41	0.13	0.43	58.27	65.46

**Figure 6:**
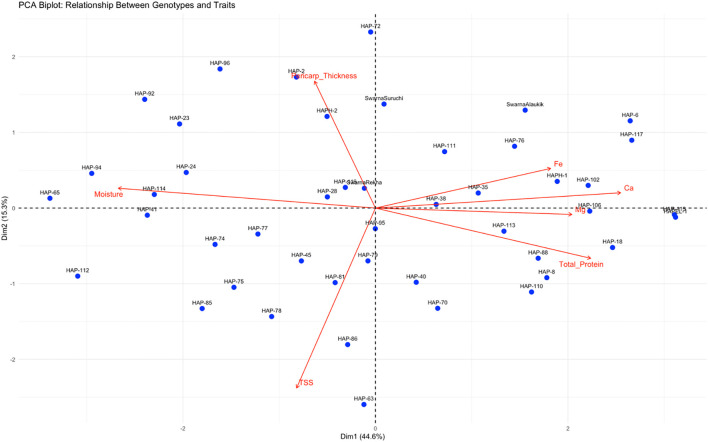
PCA Biplot depicting relationship between genotypes.

**Figure 7:**
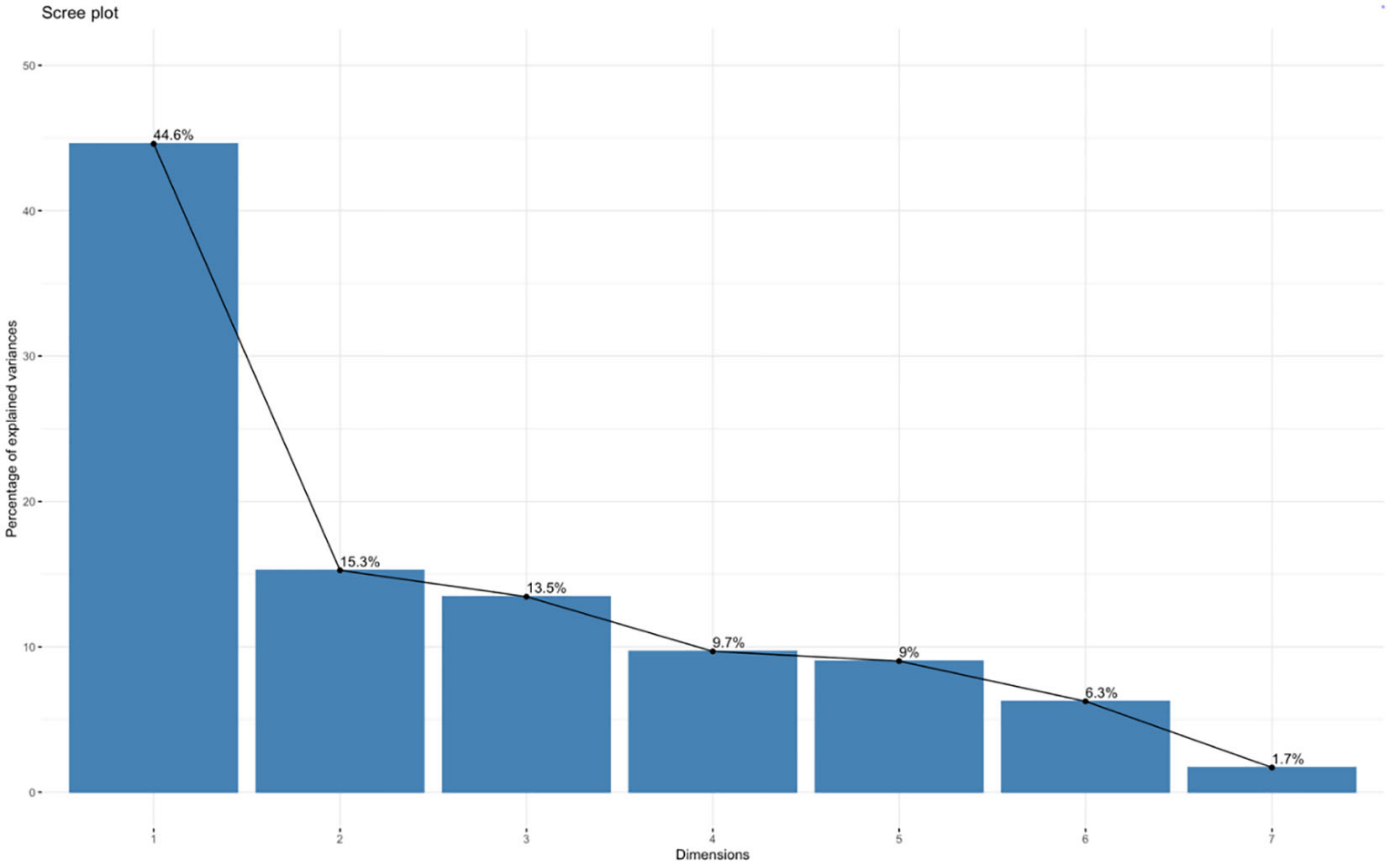
Scree plot highlighting the variance proportion.

**Figure 8:**
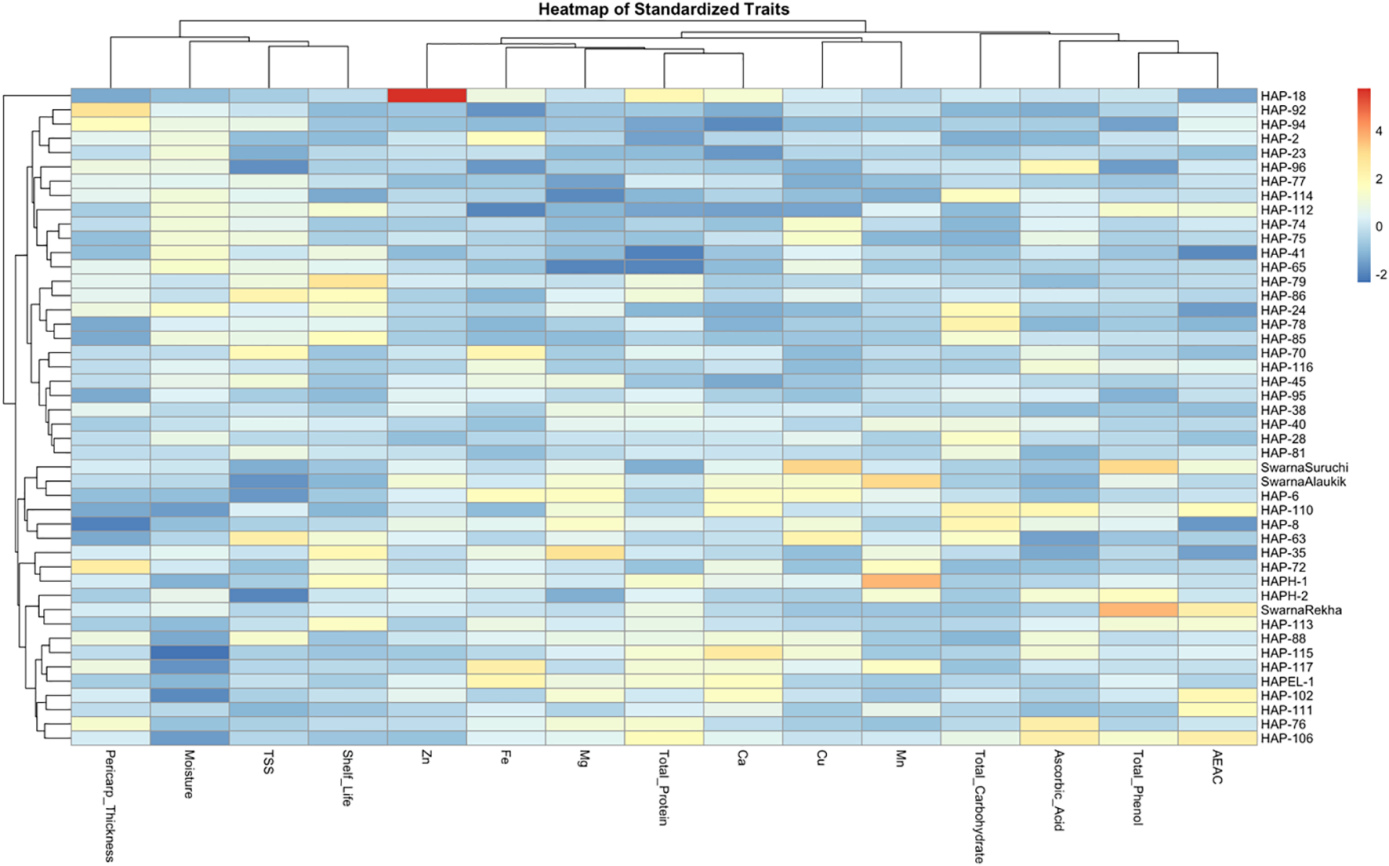
Heatmap of standardized traits.

**Figure 9:**
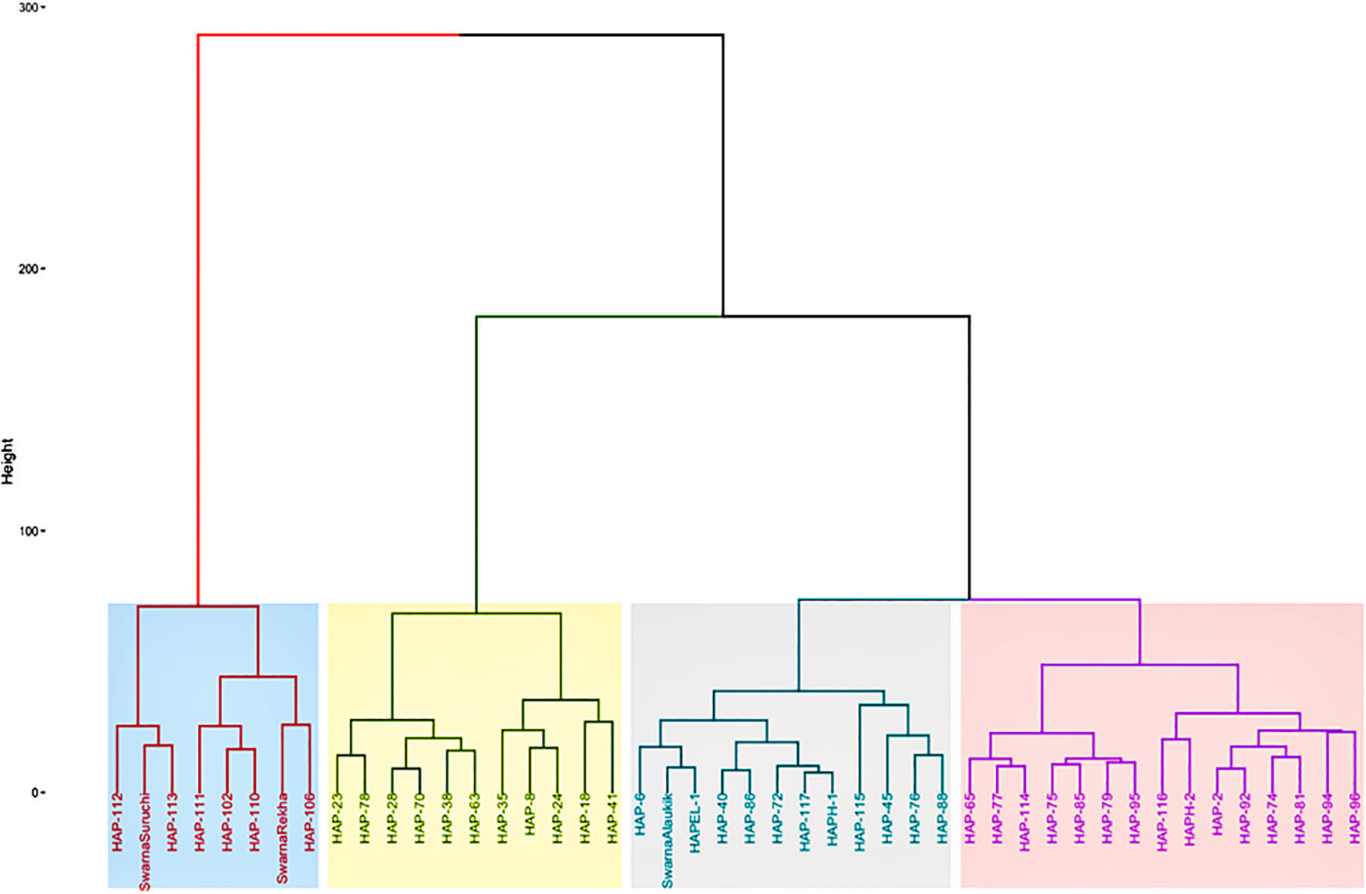
Cluster analysis of pointed gourd genotypes.

Genotypic variation was examined through Principal Component Analysis (PCA) using factoextra (Kassambara and Mundt, 2020), with the results depicted through scree plots and biplots to reveal variance distribution and genotype clustering (**Figures 6**, **7**). PCA was intentionally conducted on the normally distributed trait set, whereas non-normal traits were evaluated through Spearman’s correlation and descriptive analysis. These seven traits were selected because they passed the Shapiro–Wilk normality test (P > 0.05) and were the same traits used in the Pearson correlation matrix. For PCA interpretation, loading values with an absolute magnitude ≥ 0.40 were considered meaningful contributors. Eigenvalues, loading scores, and cumulative variance were provided in **Supplementary Tables S2 and S3**. A heatmap of standardized trait values generated using pheatmap illustrated multi-trait variation (**Figure 8**) (Kolde, 2019). Hierarchical clustering based on Euclidean distance and Ward’s method was visualized through a dendrogram using dendextend, elucidating genetic similarity patterns (**Figure 9**) (Galili, 2015). Correlation, PCA, and clustering analyses were interpreted as exploratory tools for identifying trait associations and genotype grouping patterns and were not used to infer direct biological causation.

## 4 Results

### 4.1 Analysis of variance

The analysis of variance for 15 fruit quality parameters across 46 diverse *T. dioica* genotypes is presented in **Table 1**. A significant mean sum of squares for all parameters indicates substantial variation among the pointed gourd genotypes, confirming that at least one genotype was statistically distinct from the others.

### 4.2 Distribution and variability of nutritional and biochemical traits

The violin plots (**Figure 6**) revealed variation among the 46 pointed gourd genotypes for all nutritional and biochemical traits. Wider distributions were observed for AEAC, ascorbic acid, Mg, Zn and total phenol, whereas moisture content and total protein exhibited relatively narrower distributions. Several traits, including Zn, Mn, total phenol and AEAC, showed extended tails, indicating the presence of genotypes with comparatively higher values.

### 4.3 Shelf-life of fruits

The shelf-life of fruits varied significantly among genotypes, ranging from 2.53 to 6.00 days, with an average of 3.66 days. The longest shelf-life was recorded in HAP-79 (6.00 days), followed by HAP-35 (5.33 days) and HAP-85 (5.13 days). In contrast, the shortest shelf-life was observed in HAP-114 (2.53 days), with HAP-110 (2.73 days) and Swarna Alaukik (2.73 days) showing slightly longer durations.

### 4.4 Pericarp thickness

Pericarp thickness exhibited minimal variation among genotypes, ranging from 0.30 to 0.73 cm. The highest thickness was recorded in HAP-92 (0.73 cm), followed by HAP-72 (0.70 cm), while the lowest was observed in HAP-8 (0.30 cm).

### 4.5 Fruit shape and pubescence

The distribution of fruit shapes in pointed gourd genotypes, as illustrated in the first bar plot (**Figure 9a**), shows that the most dominant fruit shape is Spindle (14 genotypes), followed by Cylindrical, Club-shaped and Elongated Spindle, each with 7 genotypes each. Spindle tapering and oval fruit shapes were observed in 5 genotypes, while Spheroid was the least common, appearing in only one genotype. The second bar plot represents the distribution of pubescence in the genotypes, where the presence of pubescence is observed in 26 genotypes, while 20 genotypes lack this trait (**Figure 9b**). This suggests a higher prevalence of pubescence among the studied pointed gourd genotypes, potentially indicating a significant morphological trait.

### 4.6 Total soluble solid

The Total Soluble Solids (TSS) content varied among the genotypes, ranging from 3.27 to 4.53 °Brix, with an average of 3.84 °Brix. The highest TSS was recorded in HAP-63 (4.53 °Brix), followed by HAP-86 (4.47 °Brix) and HAP-70 (4.40 °Brix), while HAPH-2 (3.27 °Brix) had the lowest.

### 4.7 Moisture percentage

Moisture content was determined using the gravimetric method by measuring the sample weight before and after oven drying. The moisture percentage varied slightly among genotypes, ranging from 90.33% to 95.54%, with an average of 93.46%. The highest moisture content was recorded in HAP-24 (95.54%), while HAP-115 (90.33%) had the lowest.

### 4.8 Total carbohydrate

The assessment of total carbohydrate content across pointed gourd genotypes revealed significant variation, with values ranging from 1.86 g/100 g to 9.86 g/100 g of edible fruit, indicating nearly a five-fold difference. Among the genotypes, HAP-2 recorded the lowest carbohydrate content (1.86 g/100 g), while HAP-78 exhibited the highest (9.86 g/100 g), followed closely by HAP-110 (9.78 g/100 g) and HAP-8 (9.53 g/100 g). The average carbohydrate content across all genotypes was 4.79 g/100 g, with approximately seventeen genotypes exceeding this mean value. This broad variation underscores the diversity in carbohydrate content among the studied genotypes.

### 4.9 Total protein

The total protein content in pointed gourd fruit exhibited considerable variation among the 46 genotypes analyzed. The protein content ranged from 0.63 g/100 g to 1.30 g/100 g of fresh edible fruit, with an overall average of 1 g/100 g. The highest protein content was recorded in HAP-18 (1.30 g/100 g), followed by HAP-106 (1.28 g/100 g) and HAP-76 (1.2 g/100 g), whereas HAP-41 had the lowest protein content at 0.63 g/100 g. These results highlight the substantial differences in protein levels among the genotypes studied.

### 4.10 Ascorbic acid

The ascorbic acid content among the studied pointed gourd genotypes showed considerable variation, ranging from 7.51 mg/100 g to 27.91 mg/100 g of fresh fruit weight, representing nearly a four-fold difference, as shown in the **Table 1**. The highest ascorbic acid content was recorded in genotype HAP-106 (27.91 mg/100 g), followed by HAP-76 (27.61 mg/100 g) and HAP-96 (26.10 mg/100 g). In contrast, genotype HAP-63 had the lowest content at 7.51 mg/100 g. The average ascorbic acid content across all genotypes was 15.40 mg/100 g of fresh fruit.

### 4.11 Total phenols

In this study, total phenolic content exhibited considerable variation, ranging from 17.00 to 57.76 mg GAE/100 g, representing approximately a 3.5-fold difference. This highlights significant diversity among the genotypes. The highest phenolic content was recorded in the variety Swarna Rekha (57.76 mg GAE/100 g), while the lowest was observed in genotype HAP-96 (17.01 mg GAE/100 g). The average total phenolic content across all genotypes was 29.29 mg GAE/100 g, showing significant variation.

### 4.12 Total antioxidant activity

The total antioxidant (FRAP) activity among pointed gourd genotypes exhibited substantial variation, ranging from 91.24 mg AEAC/100 g to 251.57 mg AEAC/100 g of fresh fruit, as presented in the **Table 1**. The highest antioxidant activity was observed in Swarna Rekha (251.57 mg AEAC/100 g), followed by HAP-106 (250 mg AEAC/100 g) and HAP-102 (237.97 mg AEAC/100 g), whereas the lowest value was recorded in HAP-41 (91.24 mg AEAC/100 g). The average antioxidant activity across all genotypes was 162.63 mg AEAC/100 g of fresh fruit. These results indicate significant variation in antioxidant levels among the studied genotypes.

### 4.13 Estimation of essential nutrients (Zn, Fe, Cu, Mn, Ca and Mg)

The mineral composition within pointed gourd genotypes exhibited considerable variation across different elements, including zinc, iron, copper, manganese, calcium and magnesium. The zinc concentration in the fruit ranged from 0.07 mg/100 g to 0.34 mg/100 g of fresh fruit. The highest zinc content was recorded in Swarna Alaukik (0.34 mg/100 g), whereas HAP-77 exhibited the lowest value (0.07 mg/100 g). The average zinc content across all genotypes was 0.19 mg/100 g, indicating notable diversity in zinc accumulation.

Significant variation was observed in the iron content among the genotypes, with values ranging from 0.54 mg/100 g to 2.41 mg/100 g of fruit. The highest iron concentration was found in HAP-117 (2.41 mg/100 g), while HAP-112 recorded the lowest value (0.54 mg/100 g). The overall mean iron content was 1.41 mg/100 g.

Pointed gourd exhibited relatively low levels of copper, with an average content of 0.06 mg/100 g. The copper content varied from 0.03 mg/100 g to 0.13 mg/100 g, with Swarna Suruchi (0.13 mg/100 g) showing the highest value, followed by HAP-63 (0.11 mg/100 g). The lowest copper content was recorded in HAP-112 (0.03 mg/100 g).

The manganese levels across genotypes showed a wide range, with values spanning from 0.06 mg/100 g to 0.43 mg/100 g. The highest manganese content was found in HAPH-1 (0.43 mg/100 g), followed by Swarna Alaukik (0.38 mg/100 g), while HAP-114 exhibited the lowest value (0.06 mg/100 g). The mean manganese content was 0.16 mg/100 g, highlighting significant variation.

Considerable diversity was observed in calcium levels, with values ranging between 21.44 mg/100 g and 58.27 mg/100 g. The genotype HAP-115 recorded the highest calcium content (58.27 mg/100 g), while HAP-94 had the lowest (21.44 mg/100 g). The overall mean calcium content was 37.08 mg/100 g.

Magnesium levels also varied notably across genotypes. The highest content was observed in HAP-35 (65.46 mg/100 g), followed closely by HAP-6 (53.82 mg/100 g). The mean magnesium content across all genotypes was 37.76 mg/100 g, reflecting substantial differences in magnesium accumulation.

These findings demonstrate significant variability in the mineral composition of pointed gourd genotypes, highlighting their potential nutritional value.

### 4.14 Correlation coefficient

#### 4.14.1 Correlation matrix of non-normal traits

The correlation matrix for non-normal traits (**Figure 2**) reveals several significant associations among quantitative traits in pointed gourd genotypes. Antioxidant activity (AEAC) was significantly and positively correlated with total phenol content (r = 0.376, P = 0.010; N = 46), indicating that phenolic compounds contributed to antioxidant potential. In contrast, shelf-life showed a negative correlation with AEAC (r = −0.36, P = 0.015; N = 46), suggesting a possible trade-off between antioxidant activity and post-harvest longevity. Other correlations, including those involving carbohydrate, Zn, Cu and Mn, were weak and non-significant. Interestingly, Total Carbohydrate shows a weak negative correlation with AEAC (r = -0.24) and Total Phenol (r = -0.11), suggesting that higher carbohydrate levels may correspond to lower antioxidant properties.

#### 4.14.2 Correlation matrix of normally distributed traits

The correlation matrix for normally distributed traits (**Figure 3**) revealed significant associations among moisture, protein and mineral traits. Moisture showed strong negative correlations with Ca (r = −0.817, P < 0.01) and total protein (r = −0.714, P < 0.01) and moderate negative correlations with Mg (r = −0.554, P < 0.01) and Fe (r = −0.380, P < 0.01). Total protein was positively correlated with Ca (r = 0.515, P < 0.01), Mg (r = 0.381, P < 0.01) and Fe (r = 0.354, P < 0.05). Ca also showed positive associations with Mg (r = 0.470, P < 0.01) and Fe (r = 0.437, P < 0.01). TSS and pericarp thickness showed weak and non-significant correlations with most traits.

### 4.15 Principal component analysis

The PCA biplot (**Figure 4**) summarized multivariate associations among pointed gourd genotypes and quantitative traits. Dim1 and Dim2 explained 44.6% and 15.3% of total variation, respectively. Trait vectors indicated the direction and contribution of traits in the PCA space. Moisture and total protein were positioned in opposite directions, reflecting their negative association, while Ca and Fe showed similar vector orientation, indicating positive association. TSS appeared relatively distinct from mineral-related traits. Genotypes such as HAP-6, HAP-102 and HAPH-1 were positioned near Ca and Fe vectors, whereas HAP-8, HAP-18 and HAP-110 showed proximity to total protein and Mg vectors. These patterns indicate multivariate association and should not be interpreted as evidence of causation. PCA loading values and eigenvalue statistics are provided in **Supplementary Table 2**, **3**).

The scree plot (**Figure 5**) illustrates the proportion of variance explained by each principal component (PC) in the PCA analysis of pointed gourd genotypes. The first principal component (PC1) accounts for 44.6% of the total variance, indicating that it captures the most significant variation among genotypes. The second principal component (PC2) contributes 15.3%, followed by PC3 (13.5%), PC4 (9.7%), PC5 (9.0%), PC6 (6.3%) and PC7 (1.7%). The sharp decline in variance after PC1 and PC2 suggests that these two components are the most informative for explaining genotype differences. The elbow point in the plot occurs at PC3, indicating that selecting the first three or four principal components would effectively summarize the majority of the dataset’s variability while reducing dimensionality. This confirms that most of the trait variation can be explained using a few key PCs, making them suitable for further analysis and interpretation.

The heatmap (**Figure 8**) of standardized traits presents the distribution of multiple quantitative traits across various pointed gourd genotypes, including Moisture, TSS, Shelf-life, Zn, Fe, Mg, Ca, Cu, Mn, Total Carbohydrate, Total Phenol, Ascorbic Acid, AEAC, Pericarp Thickness and Total Protein. The hierarchical clustering of genotypes, such as HAP-18, HAP-92, HAP-94, HAP-2, Swarna Suruchi and Swarna Alaukik, indicates similar trait expressions, whereas other genotypes, including HAP-102, HAP-106 and HAP-111, exhibit distinct patterns. The color gradient, where blue represents lower values and red indicates higher values, reveals variations in specific traits. Moisture and TSS levels appear to be highly variable among genotypes, while Shelf-life, Zn and Mg show moderate variation. This clustering pattern is useful for identifying genotypes with superior traits for breeding programs, particularly for enhancing nutritional and agronomic characteristics in pointed gourd improvement strategies.

The hierarchical clustering dendrogram (**Figure 7**) represents the genetic similarity among the diverse pointed gourd genotypes, with four major clusters distinguished by different colors and shaded regions. Genotypes such as HAP-112, Swarna Suruchi, HAP-113 and Swarna Rekha form a closely related group, whereas Swarna Alaukik, HAP-6 HAP-40, HAP-45, HAP-72, HAP-86, HAPH-1, HAPEL-1, HAP-72, HAP-76, HAP-117 and HAP-88 cluster separately. The clustering is based on Euclidean distance and the Ward.D2 method, with the height of branches indicating genetic divergence. The color-coded branches and filled cluster rectangles provide a clear visualization of genotypic variation, aiding in the identification of diverse parental lines for breeding and selection strategies.

## 5 Discussion

The present study revealed considerable variation in nutritional, biochemical, mineral and post-harvest traits among the evaluated pointed gourd genotypes, indicating the presence of substantial genetic diversity within the germplasm. Such diversity is an important prerequisite for crop improvement, particularly in underutilized vegetable crops where naturally occurring variation serves as the primary resource for selection and breeding (Behera and Singh, 2019; Singh et al., 2020). The wide differences observed in phenolic content, antioxidant activity, ascorbic acid, protein, carbohydrates and mineral composition suggest that genotypes vary significantly in their ability to accumulate nutrients and bioactive compounds during fruit development (Lu et al., 2014; Sarker et al., 2015; Meena et al., 2023; de Lima et al., 2024). Furthermore, the correlation, principal component and cluster analyses indicated that these traits are interconnected and influenced by underlying physiological processes related to nutrient accumulation, secondary metabolism and fruit quality (Liang et al., 2015; Durga Rao et al., 2020). These findings highlight the potential of pointed gourd germplasm for the development of nutritionally enriched cultivars with improved quality attributes.

The results revealed clear differences among pointed gourd genotypes in the accumulation of nutritional and bioactive compounds. Genotypes such as HAP-106, HAP-115, HAP-117 and HAP-18 exhibited comparatively higher levels of protein, minerals, ascorbic acid and antioxidant activity, indicating their superior nutritional quality. In contrast, some genotypes showed higher moisture content but relatively lower concentrations of these nutrients (Sarker and Oba, 2018, 2019). The strong negative correlations between moisture content and protein, calcium, magnesium and iron further support this observation (Mayer, 1997; Sarker and Oba, 2018). A possible explanation is the dilution effect, where increased water accumulation in fruit tissues reduces the concentration of nutrients on a fresh-weight basis (Mayer, 1997; Soares et al., 2019). Conversely, genotypes with lower moisture content may accumulate more dry matter, resulting in greater concentrations of proteins, minerals and other bioactive compounds (Mayer, 1997; Sarker and Oba, 2018; Soares et al., 2019). Similar relationships have been reported in several fleshy vegetable crops and highlight the importance of dry matter accumulation in determining fruit nutritional quality (Sarker and Oba, 2018, 2019; Akçura et al., 2020). The particularly strong negative association between moisture and calcium further suggests that nutrient accumulation and water content are closely related during fruit development (Bradfield and Guttridge, 1984; Mayer, 1997; Jaime-Guerrero et al., 2024). These findings highlight the importance of considering moisture content when selecting nutritionally superior genotypes. From a breeding perspective, genotypes with moderate moisture levels and higher nutrient concentrations may be valuable resources for improving the nutritional quality of pointed gourd (Scalzo et al., 2005; Bhandari et al., 2016; Kathi et al., 2024).

The positive associations observed among protein, calcium, magnesium and iron suggest that these nutritional traits are closely interconnected (Boukar et al., 2011; Akçura et al., 2020). Genotypes with higher protein content generally also exhibited higher concentrations of these minerals, indicating a coordinated pattern of nutrient accumulation (Uzun et al., 2011; Khazaei and Vandenberg, 2020). This relationship may be attributed to the important roles these minerals play in plant growth and metabolism. For example, magnesium is essential for photosynthesis and serves as a cofactor for several enzymes involved in carbon and nitrogen (Singh and Singh, 2018) while iron is required for respiration, photosynthesis and various energy-producing processes (Khan et al., 2023). Calcium contributes to cell wall development, membrane stability and nutrient transport within plant tissues (Nagar et al., 2018). Therefore, genotypes with greater efficiency in nutrient uptake, translocation and utilization are likely to accumulate higher levels of both protein and minerals (Cavalcante et al., 2019). The positive relationship among these traits is particularly important from a breeding perspective, as it suggests that selection for improved protein content may also lead to simultaneous improvement in mineral composition (Santos and Boiteux, 2013; Lopes Costa et al., 2019). Such favourable associations can facilitate the development of pointed gourd cultivars with enhanced nutritional quality through conventional breeding approaches (Bharathi and Vishalnath, 2011).

The present study revealed considerable variation among genotypes for antioxidant-related traits, including total phenols, ascorbic acid and antioxidant activity. These compounds are important components of the plant antioxidant system and play a key role in protecting cells from oxidative damage (Singh et al., 2020; Gęgotek and Skrzydlewska, 2023). In addition, they contribute significantly to the nutritional and health-promoting value of fruits and vegetables (Singh et al., 2020; Ferrari et al., 2022). The positive correlations observed between antioxidant activity, total phenols and ascorbic acid indicate that these compounds collectively contribute to the overall antioxidant potential of pointed gourd (Velioglu et al., 1998; Ji et al., 2011). Phenolic compounds are known to neutralize free radicals by donating electrons, while ascorbic acid acts as an important water-soluble antioxidant involved in maintaining cellular redox balance (Zeb, 2020; Gęgotek and Skrzydlewska, 2023). Therefore, genotypes with higher levels of phenols and vitamin C are likely to exhibit greater antioxidant activity (Rupasinghe et al., 2006). The superior performance of genotypes such as Swarna Rekha and HAP-106 for these traits suggests their potential as valuable genetic resources for improving the nutraceutical quality of pointed gourd through breeding. Interestingly, antioxidant-related traits also revealed evidence of resource allocation trade-offs within the fruit (Connor et al., 2005). Total carbohydrate content showed a negative association with antioxidant activity and phenolic concentration, suggesting competition between primary and secondary metabolism (Zrimec et al., 2025). Carbohydrates are the primary products of photosynthesis and serve as major sources of energy for growth, development and storage (García-Gómez et al., 2020). In contrast, phenolic compounds are secondary metabolites that are synthesized through specialized metabolic pathways and play important roles in plant defense and stress tolerance (Kumar et al., 2023; Sharma et al., 2026). Therefore, genotypes accumulating higher levels of carbohydrates may allocate relatively fewer resources towards the synthesis of antioxidant compounds, whereas genotypes with enhanced antioxidant potential may invest more in secondary metabolite production (Zrimec et al., 2025). Similar trends have been reported in other horticultural crops and indicate that fruit quality is influenced by the balance between primary metabolism, which supports growth and storage and secondary metabolism, which contributes to protective and health-promoting compounds (Behera and Singh, 2019; Zhang and Hao, 2020).

The positive associations of zinc and copper with total phenol and antioxidant activity suggest a close relationship between mineral nutrition and antioxidant metabolism in pointed gourd (Tamuly et al., 2013; Wang et al., 2022). Zinc showed a positive correlation with total phenols, while copper was positively associated with both phenolic content and antioxidant activity (Tamuly et al., 2013; de Lima et al., 2024). These relationships may be attributed to the important roles of these micronutrients in various physiological and biochemical processes (Sarker and Oba, 2018; Wang et al., 2024). Zinc is involved in the functioning of antioxidant enzymes that help protect plant cells from oxidative damage (Arora et al., 2002; Cabot et al., 2019), whereas copper plays a role in redox reactions and electron transport (Wang et al., 2024). Therefore, genotypes with higher concentrations of these minerals may possess a greater capacity to synthesize or maintain antioxidant compounds (Posmyk et al., 2009). Although these associations were moderate, they indicate that mineral composition may contribute to the antioxidant potential of pointed gourd fruits (Tamuly et al., 2013). This finding further highlights the interconnected nature of nutritional and nutraceutical traits and suggests that improvement of mineral content may also enhance antioxidant quality.

Shelf-life is an important trait influencing marketability, consumer acceptance and the commercial value of fresh produce (Wang and Teplitski, 2023). The observed negative association between shelf-life and antioxidant activity suggests a potential trade-off between nutritional quality and storage performance, although this relationship should be interpreted with caution as an association rather than evidence of a causal mechanism (Conversa et al., 2020). Genotypes with higher antioxidant activity often contain elevated levels of bioactive compounds such as phenols and ascorbic acid, which are linked to active metabolic processes and may accelerate physiological and biochemical changes during storage, thereby reducing shelf-life (Conversa et al., 2020; Yu et al., 2022; Tonto et al., 2023). At the same time, shelf-life was assessed through physiological loss in weight (PLW) and may also be influenced by factors such as fruit firmness, tissue structure, cuticular properties, respiration rate, moisture content and susceptibility to microbial deterioration (Kleuker and Hoffmann, 2022). The multivariate grouping of genotypes further indicated that nutritional, antioxidant, mineral and shelf-life traits were distributed differently across the evaluated germplasm, highlighting the presence of diverse and contrasting donor materials. Genotypes such as HAP-79, HAP-35 and HAP-85 exhibited relatively longer shelf-life despite comparatively lower antioxidant activity, making them promising candidates for further post-harvest evaluation and potential donor sources for improving fruit storability. Conversely, nutritionally superior genotypes including HAP-106, HAP-117, HAP-115, Swarna Alaukik and Swarna Rekha may serve as valuable preliminary donor materials for nutritional quality. The substantial variation observed among genotypes suggests that shelf-life is under genetic control and can be improved through selection (Pratta et al., 2011; Chase et al., 2024). However, validation across seasons and environments is required before firm breeding recommendations can be made. Although the physiological and biochemical mechanisms underlying extended shelf-life were not investigated in the present study, the identified genotypes represent useful genetic resources for future research aimed at improving both nutritional quality and post-harvest performance in pointed gourd through balanced breeding strategies (Pavan and Gangaprasad, 2022).

The complex relationships observed among nutritional, antioxidant, mineral and shelf-life traits were further clarified through multivariate analyses. Principal component analysis clearly separated nutrient-rich, antioxidant-rich and storage-oriented genotypes, indicating that the observed variation was associated with distinct trait combinations (Yadav et al., 2024). Similarly, hierarchical clustering grouped genotypes according to their nutritional and biochemical profiles, facilitating the identification of genetically diverse accessions with complementary attributes (Ram, 2001; Singh and Singh, 2018; Tomar et al., 2021). The distribution of genotypes in the PCA biplot suggests that nutritional quality, antioxidant potential and shelf-life characteristics are influenced by partially independent genetic factors (Svetlana et al., 2012). This finding helps explain why no single genotype excelled for all evaluated traits and highlights the challenges associated with simultaneously improving multiple quality attributes (Natalini et al., 2021). Nevertheless, the clear separation of genotypes based on trait performance provides valuable information for parent selection and breeding strategies aimed at combining superior nutritional quality with acceptable post-harvest performance.

The absence of a universally superior genotype highlights both the complexity and the opportunity associated with pointed gourd improvement (Ram, 2001; Singh et al., 2026). Rather than relying on a single elite accession, future breeding programs should focus on combining complementary attributes from multiple donor parents (Karmakar et al., 2016; Singh et al., 2020). Genotypes such as HAP-106 and HAP-18 represent valuable sources of protein and antioxidant-related traits, HAP-117 offers potential for iron enrichment, HAP-115 for calcium enhancement, Swarna Alaukik for zinc accumulation, Swarna Rekha for phenolic enrichment and HAP-79 for improved shelf-life. Strategic hybridization among these genetically diverse and physiologically contrasting parents could facilitate the development of cultivars that combine superior nutritional quality, enhanced nutraceutical value and acceptable post-harvest performance (Karmakar et al., 2016; Behera and Singh, 2019; Singh et al., 2026).

Overall, the present study demonstrated substantial genetic variability among pointed gourd genotypes for nutritional, nutraceutical, mineral and post-harvest traits. The observed negative association of moisture with protein and mineral traits may reflect a dilution effect, where higher fruit water content is associated with lower nutrient concentration on a fresh weight basis. The positive association between total phenol and antioxidant activity indicates that phenolic compounds are important contributors to antioxidant potential in pointed gourd. Similarly, positive correlations of total protein with Ca, Mg and Fe suggest partial co-accumulation of protein and mineral traits in some genotypes. The negative association between AEAC and shelf-life should be interpreted cautiously, as post-harvest longevity is influenced by multiple physiological and structural factors. Therefore, elite genotype selection was based primarily on actual trait values, supported by multivariate patterns, rather than PCA position alone. The integration of correlation analysis, principal component analysis and cluster analysis facilitated the identification of genetically diverse genotypes possessing complementary attributes. Therefore, genotypes such as HAP-106, HAP-115, HAP-117, Swarna Rekha, Swarna Alaukik and HAP-79 represent valuable genetic resources for future breeding programmes. Collectively, these findings provide a strong foundation for the development of pointed gourd cultivars with enhanced nutritional quality, improved storability and greater consumer value.

## 6 Limitations and future perspectives

While the present study provides valuable insights into the nutritional, biochemical and post-harvest diversity of pointed gourd germplasm, certain limitations should be acknowledged. The evaluation was conducted at a single location and season; therefore, the stability of the observed traits across different environments could not be assessed. In addition, genotype characterization was primarily based on phenotypic, nutritional and biochemical attributes and the incorporation of molecular markers or genomic approaches would provide a more comprehensive understanding of genetic diversity and parent selection. Shelf-life assessment was limited to physiological loss in weight (PLW), whereas additional post-harvest quality parameters such as fruit firmness, colour retention, visual quality and microbial deterioration could further improve the evaluation of storage performance. Furthermore, although correlation and multivariate analyses identified important relationships among nutritional, antioxidant, mineral and shelf-life traits, the underlying physiological and molecular mechanisms were beyond the scope of the present study. Future investigations involving multi-location trials, molecular characterization and detailed physiological analyses will be valuable for validating the stability of superior genotypes and enhancing their utilization in pointed gourd breeding programs.

## 7 Conclusion

In conclusion, the present study revealed substantial nutritional and biochemical diversity among pointed gourd genotypes, highlighting the availability of valuable genetic resources for crop improvement. Genotypes HAP-106, HAP-115, HAP-117, Swarna Rekha, Swarna Alaukik and HAP-79 exhibited superior performance for key traits, including antioxidant activity, mineral composition, nutritional quality and shelf-life, making them promising donor parents for future breeding programmes. As no single genotype combined superiority across all evaluated traits, the integration of complementary characteristics through hybridization may support future development of improved cultivars after validation across environments. Overall, these findings provide important insights into the nutritional and biochemical variability of pointed gourd germplasm and establish a strong foundation for breeding cultivars with enhanced nutritional value, improved post-harvest performance and greater consumer acceptability.

## Data Availability

The original contributions presented in the study are included in the article/**Supplementary Material**. Further inquiries can be directed to the corresponding author.
