# Supplementary material for: Exploring the nutritional riches of pointed gourd (Trichosanthes dioica Roxb.): a multivariate approach for selection of nutritionally superior genotypes for breeding programmes
Source: Front Plant Sci. 2026 Jul 15;17:1865400. doi: 10.3389/fpls.2026.1865400 (PMC13415799; doi:10.3389/fpls.2026.1865400)
Supplement: Supplementary file 1 [file Table1.docx]

| **Sl. No.** | **Genotypes**  **Supplementary Table S1: List of pointed gourd genotypes used in the study** | **Location of collection** | **Sl. No.** | **Genotypes** | **Location of collection** |
| --- | --- | --- | --- | --- | --- |
| 1 | Swarna Rekha (SR) | ICAR-RCER, Plandu, Ranchi, Jharkhand | 27 | HAP-85 | Midnapore, West Bengal |
| 2 | Swarna Suruchi (SS) | ICAR-RCER, Plandu, Ranchi, Jharkhand | 28 | HAP-86 | Faizabad, Uttar Pradesh |
| 3 | Swarna Alaukik (SA) | ICAR-RCER, Plandu, Ranchi, Jharkhand | 29 | HAP-88 | Faizabad, Uttar Pradesh |
| 4 | HAP-2 | Sabour, Bhagalpur, Bihar | 30 | HAP-92 | Plandu, Ranchi, Jharkhand |
| 5 | HAP-6 | Sabour, Bhagalpur, Bihar | 31 | HAP-94 | Plandu, Ranchi, Jharkhand |
| 6 | HAP-8 | Sabour, Bhagalpur, Bihar | 32 | HAP-95 | Plandu, Ranchi, Jharkhand |
| 7 | HAP-18 | Sabour, Bhagalpur, Bihar | 33 | HAP-96 | Plandu, Ranchi, Jharkhand |
| 8 | HAP-23 | Basti, Uttar Pradesh | 34 | HAP-102 | Plandu, Ranchi, Jharkhand |
| 9 | HAP-24 | Basti, Uttar Pradesh | 35 | HAP-106 | Plandu, Ranchi, Jharkhand |
| 10 | HAP-28 | Faizabad, Uttar Pradesh | 36 | HAP-110 | Location not known |
| 11 | HAP-35 | Sambalpur, Odisha | 37 | HAP-111 | Faizabad, Uttar Pradesh |
| 12 | HAP-38 | Sambalpur, Odisha | 38 | HAP-112 | ICAR-RCER, Plandu, Ranchi, Jharkhand |
| 13 | HAP-40 | Sambalpur, Odisha | 39 | HAP-113 | Location not known |
| 14 | HAP-41 | Sambalpur, Odisha | 40 | HAP-114 | ICAR-RCER, Plandu, Ranchi, Jharkhand |
| 15 | HAP-45 | Sambalpur, Odisha | 41 | HAP-115 | ICAR-RCER, Plandu, Ranchi, Jharkhand |
| 16 | HAP-63 | Basti, Uttar Pradesh | 42 | HAP-116 | ICAR-RCER, Plandu, Ranchi, Jharkhand |
| 17 | HAP-65 | Basti, Uttar Pradesh | 43 | HAP-117 | ICAR-RCER, Plandu, Ranchi, Jharkhand |
| 18 | HAP-70 | Faizabad, Uttar Pradesh | 44 | HAPEL-1 | Faizabad, Uttar Pradesh |
| 19 | HAP-72 | Faizabad, Uttar Pradesh | 45 | HAPH-1 | ICAR-RCER, Plandu, Ranchi, Jharkhand |
| 20 | HAP-74 | Basti, Uttar Pradesh | 46 | HAPH-2 | ICAR-RCER, Plandu, Ranchi, Jharkhand |
| 21 | HAP-75 | Faizabad, Uttar Pradesh |  |  |  |
| 22 | HAP-76 | Faizabad, Uttar Pradesh |  |  |  |
| 23 | HAP-77 | Basti, Uttar Pradesh |  |  |  |
| 24 | HAP-78 | Faizabad, Uttar Pradesh |  |  |  |
| 25 | HAP-79 | Basti, Uttar Pradesh |  |  |  |
| 26 | HAP-81 | Midnapore, West Bengal |  |  |  |

| **PC** | **Eigenvalue** | **Variance explained (%)** | **Cumulative variance explained (%)** |
| --- | --- | --- | --- |
| PC1 | 3.1223 | 44.6 | 44.6 |
| PC2 | 1.0687 | 15.27 | 59.87 |
| PC3 | 0.9418 | 13.45 | 73.33 |
| PC4 | 0.6785 | 9.69 | 83.02 |
| PC5 | 0.6317 | 9.02 | 92.04 |
| PC6 | 0.438 | 6.26 | 98.3 |
| PC7 | 0.1189 | 1.7 | 100 |

**Supplementary Table S2: Eigenvalues, Percentage of Variance Explained, and Cumulative Variance Explained by Principal Components (PCA)**

**Supplementary Table S3: Principal Component Analysis (PCA) Loading Scores of Variables Across Principal Components**

| **Trait** | **PC1** | **PC2** | **PC3** | **PC4** | **PC5** | **PC6** | **PC7** |
| --- | --- | --- | --- | --- | --- | --- | --- |
| Moisture | -0.51 | 0.09 | -0.23 | -0.21 | -0.11 | 0.18 | 0.77 |
| Total Protein | 0.43 | -0.22 | 0.26 | 0.02 | 0.40 | 0.67 | 0.29 |
| TSS | -0.16 | -0.78 | 0.41 | -0.33 | -0.10 | -0.27 | 0.07 |
| Fe | 0.35 | 0.17 | -0.25 | -0.88 | 0.07 | -0.05 | -0.08 |
| Ca | 0.49 | 0.07 | 0.01 | 0.20 | 0.15 | -0.63 | 0.55 |
| Mg | 0.39 | -0.03 | 0.04 | 0.05 | -0.89 | 0.20 | 0.11 |
| Pericarp Thickness | -0.12 | 0.55 | 0.80 | -0.17 | -0.05 | -0.05 | 0.05 |

| **Trait 1**  **Supplementary Table S4: Pearson's Correlation Coefficients (r), P-values, and Significance Levels Among the Studied Traits** | **Trait 2** | **Pearson's Correlation Coefficients (r)** | **P-value** | **Significance** |
| --- | --- | --- | --- | --- |
| Moisture | Moisture | 1 | 0 | ** |
| Total Protein | Moisture | -0.714 | 2.505E-08 | ** |
| TSS | Moisture | 0.129 | 0.3923 | ns |
| Fe | Moisture | -0.38 | 0.009239 | ** |
| Ca | Moisture | -0.817 | 4.294E-12 | ** |
| Mg | Moisture | -0.554 | 0.00006544 | ** |
| Pericarp Thickness | Moisture | 0.101 | 0.5053 | ns |
| Moisture | Total Protein | -0.714 | 2.505E-08 | ** |
| Total Protein | Total Protein | 1 | 0 | ** |
| TSS | Total Protein | -0.035 | 0.8194 | ns |
| Fe | Total Protein | 0.354 | 0.01577 | * |
| Ca | Total Protein | 0.515 | 0.0002462 | ** |
| Mg | Total Protein | 0.381 | 0.009055 | ** |
| Pericarp Thickness | Total Protein | -0.118 | 0.4331 | ns |
| Moisture | TSS | 0.129 | 0.3923 | ns |
| Total Protein | TSS | -0.035 | 0.8194 | ns |
| TSS | TSS | 1 | 0 | ** |
| Fe | TSS | -0.214 | 0.1542 | ns |
| Ca | TSS | -0.268 | 0.07227 | ns |
| Mg | TSS | -0.133 | 0.3783 | ns |
| Pericarp Thickness | TSS | -0.039 | 0.797 | ns |
| Moisture | Fe | -0.38 | 0.009239 | ** |
| Total Protein | Fe | 0.354 | 0.01577 | * |
| TSS | Fe | -0.214 | 0.1542 | ns |
| Fe | Fe | 1 | 0 | ** |
| Ca | Fe | 0.437 | 0.002378 | ** |
| Mg | Fe | 0.34 | 0.02085 | * |
| Pericarp Thickness | Fe | -0.118 | 0.4367 | ns |
| Moisture | Ca | -0.817 | 4.294E-12 | ** |
| Total Protein | Ca | 0.515 | 0.0002462 | ** |
| TSS | Ca | -0.268 | 0.07227 | ns |
| Fe | Ca | 0.437 | 0.002378 | ** |
| Ca | Ca | 1 | 0 | ** |
| Mg | Ca | 0.47 | 0.0009743 | ** |
| Pericarp Thickness | Ca | -0.152 | 0.3121 | ns |
| Moisture | Mg | -0.554 | 0.00006544 | ** |
| Total Protein | Mg | 0.381 | 0.009055 | ** |
| TSS | Mg | -0.133 | 0.3783 | ns |
| Fe | Mg | 0.34 | 0.02085 | * |
| Ca | Mg | 0.47 | 0.0009743 | ** |
| Mg | Mg | 1 | 0 | ** |
| Pericarp Thickness | Mg | -0.118 | 0.4359 | ns |
| Moisture | Pericarp Thickness | 0.101 | 0.5053 | ns |
| Total Protein | Pericarp Thickness | -0.118 | 0.4331 | ns |
| TSS | Pericarp Thickness | -0.039 | 0.797 | ns |
| Fe | Pericarp Thickness | -0.118 | 0.4367 | ns |
| Ca | Pericarp Thickness | -0.152 | 0.3121 | ns |
| Mg | Pericarp Thickness | -0.118 | 0.4359 | ns |
| Pericarp Thickness | Pericarp Thickness | 1 | 0 | ** |

P ≤ 0.05 and P ≤ 0.01, as *(significant) and ** (highly significant), respectively; ; ns: non-significant; N=46

**Supplementary Table S5: Spearman's Correlation Coefficients (ρ), P-values, and Significance Levels Among the Studied Traits**

| **Trait 1** | **Trait 2** | **Spearman's ρ** | **P-value** | **Significance** |
| --- | --- | --- | --- | --- |
| Total Carbohydrate | Total Carbohydrate | 1 | 0 | ** |
| Total Phenol | Total Carbohydrate | -0.111 | 0.4646 | ns |
| AEAC | Total Carbohydrate | -0.241 | 0.1069 | ns |
| Ascorbic Acid | Total Carbohydrate | 0.028 | 0.8544 | ns |
| Shelf Life | Total Carbohydrate | 0.109 | 0.4707 | ns |
| Zn | Total Carbohydrate | 0.02 | 0.8958 | ns |
| Cu | Total Carbohydrate | -0.125 | 0.4095 | ns |
| Mn | Total Carbohydrate | -0.134 | 0.376 | ns |
| Total Carbohydrate | Total Phenol | -0.111 | 0.4646 | ns |
| Total Phenol | Total Phenol | 1 | 0 | ** |
| AEAC | Total Phenol | 0.376 | 0.01012 | * |
| Ascorbic Acid | Total Phenol | 0.241 | 0.1072 | ns |
| Shelf Life | Total Phenol | -0.038 | 0.8004 | ns |
| Zn | Total Phenol | 0.282 | 0.05746 | ns |
| Cu | Total Phenol | 0.235 | 0.1155 | ns |
| Mn | Total Phenol | 0.083 | 0.5857 | ns |
| Total Carbohydrate | AEAC | -0.241 | 0.1069 | ns |
| Total Phenol | AEAC | 0.376 | 0.01012 | * |
| AEAC | AEAC | 1 | 0 | ** |
| Ascorbic Acid | AEAC | 0.211 | 0.1591 | ns |
| Shelf Life | AEAC | -0.357 | 0.01499 | * |
| Zn | AEAC | -0.109 | 0.4699 | ns |
| Cu | AEAC | -0.099 | 0.5134 | ns |
| Mn | AEAC | -0.025 | 0.8672 | ns |
| Total Carbohydrate | Ascorbic Acid | 0.028 | 0.8544 | ns |
| Total Phenol | Ascorbic Acid | 0.241 | 0.1072 | ns |
| AEAC | Ascorbic Acid | 0.211 | 0.1591 | ns |
| Ascorbic Acid | Ascorbic Acid | 1 | 0 | ** |
| Shelf Life | Ascorbic Acid | -0.187 | 0.2134 | ns |
| Zn | Ascorbic Acid | -0.157 | 0.2981 | ns |
| Cu | Ascorbic Acid | -0.071 | 0.6413 | ns |
| Mn | Ascorbic Acid | -0.234 | 0.1179 | ns |
| Total Carbohydrate | Shelf Life | 0.109 | 0.4707 | ns |
| Total Phenol | Shelf Life | -0.038 | 0.8004 | ns |
| AEAC | Shelf Life | -0.357 | 0.01499 | * |
| Ascorbic Acid | Shelf Life | -0.187 | 0.2134 | ns |
| Shelf Life | Shelf Life | 1 | 0 | ** |
| Zn | Shelf Life | -0.104 | 0.4924 | ns |
| Cu | Shelf Life | -0.124 | 0.4105 | ns |
| Mn | Shelf Life | 0.052 | 0.7308 | ns |
| Total Carbohydrate | Zn | 0.02 | 0.8958 | ns |
| Total Phenol | Zn | 0.282 | 0.05746 | ns |
| AEAC | Zn | -0.109 | 0.4699 | ns |
| Ascorbic Acid | Zn | -0.157 | 0.2981 | ns |
| Shelf Life | Zn | -0.104 | 0.4924 | ns |
| Zn | Zn | 1 | 0 | ** |
| Cu | Zn | 0.217 | 0.1472 | ns |
| Mn | Zn | 0.215 | 0.1505 | ns |
| Total Carbohydrate | Cu | -0.125 | 0.4095 | ns |
| Total Phenol | Cu | 0.235 | 0.1155 | ns |
| AEAC | Cu | -0.099 | 0.5134 | ns |
| Ascorbic Acid | Cu | -0.071 | 0.6413 | ns |
| Shelf Life | Cu | -0.124 | 0.4105 | ns |
| Zn | Cu | 0.217 | 0.1472 | ns |
| Cu | Cu | 1 | 0 | ** |
| Mn | Cu | 0.122 | 0.4179 | ns |
| Total Carbohydrate | Mn | -0.134 | 0.376 | ns |
| Total Phenol | Mn | 0.083 | 0.5857 | ns |
| AEAC | Mn | -0.025 | 0.8672 | ns |
| Ascorbic Acid | Mn | -0.234 | 0.1179 | ns |
| Shelf Life | Mn | 0.052 | 0.7308 | ns |
| Zn | Mn | 0.215 | 0.1505 | ns |
| Cu | Mn | 0.122 | 0.4179 | ns |
| Mn | Mn | 1 | 0 | ** |

ρ = Spearman's correlation coefficient; ns = non-significant (P > 0.05); * = significant at P ≤ 0.05; ** = significant at P ≤ 0.01.; N=46
